# The Relationship between Functional Promoter Variants of Macrophage Migration Inhibitory Factor and Endometriosis

**DOI:** 10.22074/cellj.2021.6858

**Published:** 2020-04-22

**Authors:** Zahra Chekini, Maryam Shahhoseini, Reza Aflatoonian, Parvaneh Afsharian

**Affiliations:** 1 Department of Biology, Science and Research Branch, Islamic Azad University, Tehran, Iran; 2 Department of Endocrinology and Female Infertility, Reproductive Biomedicine Research Center, Royan Institute for Reproductive Biomedicine, ACECR, Tehran, Iran; 3 Reproductive Epidemiology Research Center, Royan Institute for Reproductive Biomedicine, ACECR, Tehran, Iran; 4 Department of Genetics, Reproductive Biomedicine Research Center, Royan Institute for Reproductive Biomedicine, ACECR, Tehran, Iran

**Keywords:** Endometriosis, Gene Expression, Haplotype, Macrophage Migration Inhibitory Factor, Polymorphism

## Abstract

**Objective:**

Endometriosis is a common gynecological and inflammatory disorder. Macrophage migration inhibitory factor (MIF) is a key pro-inflammatory cytokine that is secreted by accumulated active macrophages in ectopic endometrial tissues. Two promoter polymorphisms of *MIF [-794(CATT)^5–8^/-173G/C]* were identified to susceptibility and severity of several immune and inflammatory diseases. We aimed to evaluate the possible association between *MIF* promoter polymorphisms and susceptibly to endometriosis and its corolation with mRNA level.

**Materials and Methods:**

This case-control study was performed in Royan Institute from 2015 to 2017. Polymorphisms were evaluated in 106 endometriosis patients and 110 controls. For 17 endometrioma tissues, gene expression studies were conducted during secretory phase of menstrual cycle. Restriction fragment length polymorphism (RFLP) analysis was performed to determine -173G/C polymorphism and -794(CATT)^5–8^ were detected by sequencing. Quantitative polymerase chain reaction (Q-PCR) was carried out to determine MIF expression level.

**Results:**

Homozygote of CATT_7_ was observed only in endometriosis whilst we did not detect the significant allele and genotype variation in both groups. The homozygotes for -794(CATT)^5–8^ and -173G/C polymorphisms were obtained to estimate the haplotype frequencies. Significantly higher haplotype frequencies were observed for CATT_5_/G in controls [global P value=0.044]. Additionally, the CATT_5_/C and CATT_7_/G haplotypes were not detected in any groups. Expression level of mRNA in ectopic tissue of endometriosis patients with CATT_6,7_/CC haplotype, were significantly higher compared to other haplotypes including CATT_5,5_/GG (2.91 fold, P=0.007), CATT_5,5_/GC (2.48 fold, P=0.047) and CATT_6,6_/GG (2.08 fold, P=0.046).

**Conclusion:**

We report, for the first time, a strong linkage between the decreased repetition of CATT and G allele in control and CATT_6_/C and CATT_7_/C haplotypes in endometriosis patients. Increased MIF expression is affected by genetic variants in the MIF promoter in ectopic endometrial tissues. This promoter haplotype might play an important role in the development and establishment of endometriosis.

## Introduction

Endometriosis is an inflammatory, estrogen-dependent disease that is characterized by presence of ectopic endometrial-like tissue outside of the uterine cavity (1, 2). Endometriosis is associated with pelvic pain and infertility in most patients (2). Several theories explained the pathogenesis of the condition (3), but the Sampson’s hypothesis is the most-widely accepted one which suggested a retrograde movement of endometrial cells via the fallopian tubes into the peritoneal cavity during menstruation (4). It seems that approximately 90% of women possess retrograde menstruation; however, refluxed endometrial cells are usually cleared by macrophages, natural killer (NK) cells, and lymphocytes but in endometriosis patients, a combination of impaired immunological clearance and aberrant cytokine expression interferes with clearance of the ectopic lesions leading to establishment and development of the disease (5). The impaired immune response is related to reduced cytotoxic activity of NK cells, increased number of T cells and accumulation of activated macrophages (6, 7).

Macrophage migration inhibitory factor (MIF) is a pleiotropic pro-inflammatory cytokine (8, 9) that is produced by T lymphocytes and accumulative macrophages and activates several molecular pathways in the ectopic endometrial tissue (10). The MIF-induced extracellular mitogen-activated (MAP) kinase pathway causes an increase in prostaglandin E2 (PGE2) and estrogen, also negatively regulates p53 and promotes apoptosis; thus, its inhibition may enhance proliferation (11, 12). Several studies as well as our group suggested that mRNA and plasma levels of MIF are increased in the ectopic and eutopic tissues of endometriosis patients (12-15) but no genetic variation was described.

The *MIF* gene is located in the chromosome 22q11.2 region and consists of three exons (16). Polymorphisms with potential functional relevance were also identified in the *MIF* promoter (17); a single nucleotide polymorphism (SNP) at position -173G/C (rs755622) and a short tandem repeat polymorphism (STRP), -794 (CATT)_5–8_ (rs5844572), were shown by several meta-analyses to increase susceptibility some immune and inflammatory diseases and their severity (18-21).

Since endometriosis is an inflammatory disorder and increased levels of *MIF* are observed in ectopic tissues, we aimed to evaluate *MIF* promoter variations that could be involved in development of endometriosis and susceptibility towards this disorder. Also, *MIF* mRNA expression levels in ectopic tissues from patients with endometriosis who carried different genotypes for the two promoter variations were determined.

## Materials and Methods

### Subjects

This case-control study was approved by Ethics Committee of Royan Institute (No.EC/91/1137) and each participant signed an informed consent form. The stage of endometriosis lesions was categorized based on the revised classification of the American Fertility Society (rAFS). In the current study, 106 patients with diagnosed endometriosis, who had undergone laparoscopy from 2015 to 2017 and had endometrioma cysts confirmed by histological tests, were enrolled. The endometrioma tissues were collected during laparoscopic surgery in the secretory phases of menstrual cycle (days 16-19). The 110 controls were recruited from subjects who were not diagnosed with endometriosis, underwent diagnostic laparoscopy or fertile women with no sign of endometriosis in Doppler ultrasonography. None of the participants had endometrial hyperplasia, neoplasia, or inflammatory and autoimmune disorders, and none of them were receiving anti-inflammatory or hormonal medication for at least 3 months before laparoscopy. The subjects’ age was between 20 and 40 years old and the control individuals were matched with the endometriosis patients in terms of body mass index (BMI).

### Identification of the MIF polymorphisms

#### DNA extraction

DNA was extracted from whole blood anticoagulated with ethylenediamine tetraacetic acid (EDTA)-2Na. Patients’ genomic DNA was extracted by using the Gene All® kit (Korea), according to the manufacturer’s instructions. Salting-out method was used to obtain the controls’ genomic DNA.

### Polymerase chain reaction reactions

The polymerase chain reaction (PCR) was used to amplify the studied fragments of MIF gene. The PCR included a hot start at 95˚C for 5 minutes, followed by 35 PCR cycles, each including denaturation for 30 seconds at 94˚C, primer annealing (depending on the primer pairs) for 30 seconds, and extension for 60 seconds at 72˚C. A final extension step was conducted at 72˚C for 10 minutes.

### Genotyping of the -173G/C polymorphism

Restriction fragment length polymorphism (RFLP) was performed to detect -173G/C SNP. PCR was used to amplify a 303 bp fragment.

Sense primer was:

5´- CCT-CCT-GGC-GAC-TAA-CAT-CGG-TGA-CT-3´

and the anti-sense primer was:

 5´- TAC-GTG-CCT-GAC-TTC-TCG-GAC-ACC-ACT -3´

The annealing temperature was set at 63˚C. The resulting fragment was digested using AluI restriction endonuclease (Fermentase Biolabs, MA, USA) for 15 minutes at 37˚C, and the digested fragments were resolved using 1.7% agarose gel stained with ethidium bromide, and visualized using Molecular Imager® Gel Doc™ XR+ (BioRad, California, USA) under ultraviolet (UV) light. The GG genotype revealed a single band (303 bp) because no cutting site for this enzyme, while two small PCR fragments containing 98 and 205 bp represent CC genotype. The RFLP pattern for heterozygous GC was characterized using the following 3 bands: 303, 205 and 98bp. More than 10% of the samples with different genotypes, were randomly selected to be sequenced (Macro gen, Geumcheon-gu, Korea) to confirm the genotypes obtained by PCR- RFLP method.

### Microsatellite typing

Oligonucleotide primers (sense primer:

5´-TAT- GGA-TTG-CAC-CTA-TCA-GAG-ACC-3´

 and anti-sense primer:

 5´-TCT-CAT-AGA-GCC-CTT-GGT-GAAT-3´),

 were designed to amplify a 250 bp segment of the -794(CATT)_5-8_ promoter region. The annealing temperature of PCR cycle was 58˚C. Purified PCR products of -794(CATT)_5-8_ and ORF region were sequenced using an ABI automated DNA sequencer (Macro gen, Geumcheon-gu, Korea).

### Real-time fluorescent quantitative polymerase chain reaction

Endometriotic tissues were collected from 17 endometrioma lesions in women who were genotyped for the *MIF* promoter. The RNA was isolated using TRIzol (TRI<sup>®</sup> reagent, Sigma-Aldrich, St Louis, MO, USA) based on the manufacturer’s instructions. cDNA was amplified by One-Step Reverse transcriptase (RT)-PCR using a transcriptase kit (Fermentase Biolabs, Ipswich, MA, USA) in the presence of random hexamers. The reaction was incubated at 25˚C for 5 minutes, 42˚C for 60 minutes, and 70˚C for 5 minutes. The RT-PCR products were run on agarose gel. Quantitative real-time PCR was performed in an ABI 7000 Thermal Cycler (Applied Biosystems, Foster City, CA, USA). Each standard PCR reaction contained 2 μl cDNA templates, 1 μl of each primer (final concentration, 0.1 mmol/L), and 10 μl SYBR Green PCR Master Mix (Applied Biosystems, Foster City, CA, USA) containing Taq DNA polymerase buffer, deoxynucleotide triphosphate mix, SYBR green I, MgCl2, and Taq DNA polymerase. After denaturation (for 4 minutes at 95˚C), amplification and quantification were repeated 40 times (10 seconds at 95˚C, 30 seconds at 60˚C, and 30 seconds at 72˚C). The primer pairs (in the 5’-3’direction) used for human *MIF*:

[(sense:

5´-AGA-ACC-GCT-CCT-ACA-GCA-AG-3´

antisense: ]

5´-GAG-TTG-TTC-CAG-CCC-ACA-TT-3´

and amplicon size: 121bp) and

(sense:

5´-CAA-GAT-CAT-TGC-TCC-TCC-TG-3´

and antisense:

5´-ATC-CAC-ATC-TGC-TGG-AAG-G-3´

 and amplicon size: 90bp)

for *β-Actin* were described in our previous study (15). The specificity of the PCR product was estimated by melting curve analysis. All experiments were carried out in triplicate and the relative expression was evaluated using the 2^-ΔΔCt^ method.

### Statistical analysis

Comparison of Hardy-Weinberg equilibrium test results was made and allelic and genotype distributions were compared between endometriosis patients and controls using the Pearson’s Chi-square analysis by SHEsis (http://analysis.bio-x.cn) (22). The homozygotes for -794(CATT)_5-8_ and -173G/C were evaluated by SHEsis for haplotype distribution, odds ratios (ORs) and 95% confidence interval (CI) (23). For haplotype analyses, scale significantly was considered global P values<0.05. Clinical features of endometriosis patients were studied and comparisons of *MIF* mRNA levels in endometrioma lesions were made using one-way ANOVA and the Tukey’s test, and the results were presented as mean ± standard deviation (SD). Differences were considered statistically significant at P<0.05. All statistical analyses were performed using Statistical Package for the Social Sciences (SPSS Inc., version 22, Chicago, IL, USA) software.

## Results

### Characteristics of the study population

All endometriosis participants had severe endometriosis (stage III and IV). The two groups matched on age with a mean distribution of 31 ± 3.74 and 31.75 ± 3.52 years in endometriosis patients and controls, respectively. BMI has not significant difference between both groups (25.2 ± 3.52 and 24.8 ± 3.8 Kg/m2 in endometriosis and control groups, respectively). All participants in the current study have a regular menstruation cycle.

### Promoter polymorphisms and haplotype study

Genotype and allele frequencies were in Hardy– Weinberg equilibrium in both groups (P>0.05). As shown in Table 1, the CATT8 allele was not detected neither in endometriosis patients nor in controls. Homozygote of CATT7 was observed only in endometriosis whilst CATT6 and CATT7 alleles were more prevalent in endometriosis patients and CATT5 allele was more prevalent in controls but these differences were not significant (P>0.05). Therefore, at the first step, we studied the -794 (CATT)_5-8_ polymorphisms (Table 1) and found that 53 out of 106 endometriosis patients and 53 subjects out of 110 controls were homozygous. In order to assess the effect of simultaneous occurrence of two promoter polymorphisms (-794(CATT)_5-8_ and -173G/ C), we continued to evaluate the frequencies of -173G/ C polymorphism in 53 endometriosis patients and 53 controls with homozygous genotypes of -794(CATT)_5-8_ polymorphism (Table 2). The data obtained from -173G/ C genotyping in these patients were used for this analysis extracted from our previous related study (24). Finally, considering the results presented in Tables 1 and 2, samples from 43 endometriosis patients and 46 controls who were homozygotes for both -794(CATT)_5-8_ and -173G/C polymorphisms, were investigated to estimate the haplotype frequencies (Table 3). Since the purpose of this study was run a haplotype analysis, it was essential to eliminate heterozygous subjects. The homozygous for -794(CATT)_5-8_ polymorphism was more frequently accompanied by the GG genotype of the -173GC in both groups. With respect to haplotypic frequencies, CATT5/G haplotype was significantly more frequent in controls [global test P=0.044]. We observed similar distributions for CATT_6_/G haplotype in both groups; however, the carriage of the CATT_6_/C and CATT_7_/C haplotypes was associated with higher endometriosis susceptibility, but the difference was not statistically significant (P>0.05). Additionally, the CATT_5_/C and CATT_7_/G haplotypes were not detected in any group (Table 3). Therefore, strong linkage between decreased repetition of CATT and G allele was detected.

**Table 1:** Distribution of genotype and allele frequency of MIF -794(CATT)5-8 in endometriosis patients and controls


Variant	Endometriosis	Controls	P value
	n=106	n=110	

-794(CATT)_5-8_ genotype			
5/5^*^	3 (2.8)	9 (8.2)	0.339
5/6	40 (37.8)	45 (40.9)	
6/6^*^	49 (46.2)	44 (40)	
6/7	13 (12.3)	12 (10.9)	
7/7^*^	1 (0.9)	0	
-794(CATT)_5-8_ allele			
5	46 (21.7)	63 (28.6)	0.227
6	151 (71.2)	145 (65.9)	
7	15 (7.1)	12 (5.5)	


Data are presented as n (%). *; Homozygote subjects identified for further analysis (in total 53 endometriosis patients and 53 controls were included in this analysis).

**Table 2:** Distribution of genotype and allele frequency of MIF -173G/C between -794(CATT)5-8 homozygotes in endometriosis patients and controls


Variant	Endometriosis	Controls	P value
	n=53	n=53	

-173G/C genotype			
GG^*^	39 (73.6)	45 (84.9)	0.251
GC	10 (18.9)	7 (13.2)	
CC^*^	4 (7.5)	1 (1.9)	
-173G/C allele			
G	88 (83)	97 (91.5)	0.063
C	18 (17)	9 (8.5)	


Data are presented as n (%). *; Homozygote subjects identified for further analysis.

**Table 3:** Distribution of MIF -794(CATT)5-8 and -173G/C haplotype in endometriosis patients and controls*


Haplotype	Endometriosis	Controls	P value	OR [95% CI]
	n=43	n=46		

5G	6 (7)	16 (17.4)	0.04	0.365 [0.136-0.983]
5C	0	0	-	-
6G	72 (83.7)	74 (80.4)	0.352	1.459 [0.656-3.246]
6C	6 (7)	2 (2.2)	0.113	3.462 [0.697-17.646]
7G	0	0	-	-
7C	2 (2.3)	0	-	-


Data are presented as n (%). *; Homozygous of -794(CATT)5-8 and -173G/C were included in haplotype frequencies assessment, OR; Odds ratios, and CI; Confidence interval.

### Expression of *MIF* correlates with haplotype of *MIF* promoter

**Fig.1:**
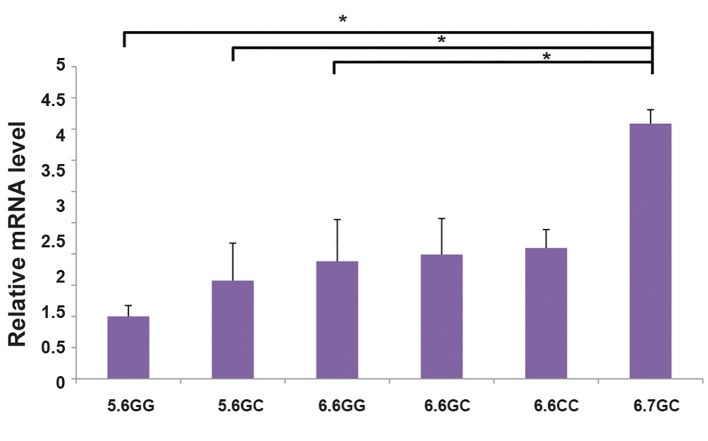
Analysis of MIF mRNA levels and evaluation of the promoter haplotype with different MIF -794 CATT genotypes (5/5, 5/6, 6/6, and 6/7) together with -173 (GG/GC/CC). Comparison between groups was made by analysis of means ± SEM. Bars show the mean and SEM of experiment performed in triplicate. *; P<0.05.

To evaluate the promoter haplotype -173G/C and -794(CATT)_5-8_ and function with *MIF* mRNA expression, we assessed the levels of mRNA using fluorescent quantitative polymerase chain reaction (FQ-PCR) in 17 patients with endometrioma who were also genotyped for promoter polymorphisms. The data were normalized against the mRNA level of the CATT_5_/G samples. We found an interaction between the -173C and more copies of repetitions of CATT in ectopic endometriotic tissues. Promoter activity and subsequent expression of mRNA in ectopic tissue of patients with CATT_6,7_/CC haplotype were significantly higher compared to other haplotypes including CATT_5,5_/GG (2.91 fold, P=0.007), CATT_5,5_/ GC (2.48 fold, P=0.047) and CATT6,6/GG (2.08 fold, P=0.046). However, the higher transcriptional activity in individuals carrying CATT_6,6_/GC (2.07 fold, P=0.113) and CATT_6,6_/CC (2.01 fold, P=0.130) was not significantly different from those of subjects carrying CATT_6,7_/CC haplotype (Fig.1).

## Discussion

Considering the importance of the *MIF* gene in promoting the inflammatory processes and establishment of ectopic tissues, we investigated possible associations between genetic variants of *MIF* promoter and susceptibility to endometriosis. The results revealed that genetic variants of *MIF*, including the 7 repetitions of the CATT STR in homozygote, were only detected in subjects with endometriosis. Exceptionally, the CATT_8_ allele, which is a rare allele, was not detected in this study.

Based on our results, the -173C allele is more common in patients carrying -794(CATT)5-8 homozygotes suggesting it as a risk factor for endometriosis. This finding was confirmed by haplotype analysis which revealed that CATT_6_/C and CATT7/C haplotypes can be considered as moderate risk factors and CATT_5_/G has an almost protective effect against endometriosis. Additionally, the CATT_5_/C and CATT7/G haplotypes were not detected in any of the groups.

Geographic variation in -794 STRP also exists and farther from Kenya and Zambia, the frequency distribution of MIF-794 STRP with 5 repeats was lower than other genotypes (25), while in white and Northeast Asian populations, the 6-repeat allele was predominant (26). The western populations with short tandem repeat of MIF CATT_5_ were less susceptible to autoimmune inflammation (27) and in Northeast Asian populations, the 6-repeat allele was predominant (26). In our study, the most frequent CATT5 allele showed protective properties against endometriosis, which is similar to the effect observed in the Asian population, whereas the 8- repeat of CATT allele was not detected in this study.

Donn et al. (28), for the first time, reported that -173C *MIF* variation is related to inflammatory disorders. Also, Baugh et al. (29), first proposed that -794CATT has five to eight-repeat units and found that short CATT repetitions have a protective effect on rheumatoid arthritis (RA) and suggested that CATT_7_/C haplotype is related to increased MIF level. Up to now, several meta-analyses investigated possible associations between -173C and CATT_7_/C, and inflammatory and autoimmune disorders such as tuberculosis (TB), juvenile rheumatoid arthritis (JRA), inflammatory bowel disease (IBD) and cancers (18-21, 30, 31). Another study evaluated associations between haplotype promoter and MIF expression level and demonstrated that the 7-repeat at the -794CATT and C allele at the -173 G/C position (7C haplotype) are related to increased *MIF* expression in RA (19). Also, we previously showed the over-expression of *MIF* in ectopic tissues from endometriosis patients (15). The presence of C in the -173 promoter region introduces an AP-4 (activating enhancer binding protein 4) transcription factor binding site (28). AP-4 plays an important role in cellular function by regulation of genes involved in cell growth, survival, immune response and angiogenesis (32). Therefore, the presence of C allele in this region increases the tendency of DNA to bind AP-4 transcription factor.

At the promoter region, the CATT repetition contains several identification regions pituitary-specific factor 1 (Pit-1) binding sites. Pit-1 is a transcription factor in neuroendocrine and mononuclear cells (33, 34). Also, recent studies revealed that inverted CCAAT box binding protein of 90 kDa (ICBP90) is the transcription factor required for interactions between *MIF* microsatellite in several immune system cells and synovial fibroblasts (35). Pit-1 and ICBP90 regulate *MIF* promoter function that is dependent on the length of tandem repetition (34, 35). This may be the reason for the association between the *MIF* CATT^7^/C genotype (haplotype) and the susceptibility towards endometriosis, because this haplotype causes the simultaneous presence of AP-4, Pit-1 and ICBP90 transcription factors and consequently enhances *MIF* promoter activity. The results showed an increased expression of *MIF* mRNA in individuals with 6C compared to those with 5G and 7C haplotypes. Thus, observed simultaneous attendance of longer CATT repeats at -794 and the -173C allele in the gene was associated with elevated MIF production and correlated with increased risk of endometriosis. This was confirmed in haplotype analysis which revealed that CATT_5_/G has strongly protective effect against endometriosis. Taken together, our findings indicate that increment of *MIF* expression is associated with genetic variants of *MIF* promoter in ectopic endometriotic tissues.

Increased MIF activates a cascade of events and strongly stimulates cyclooxygenase 2 (COX2) and prostaglandin E2 (PGE2) expression. This finally leads to increased local synthesis of estrogen in ectopic tissues, which is involved in maintenance and progression of endometriosis (36). Thus, increased MIF is associated with facilitated growth, angiogenesis and development of endometriosis tissue (11, 37).

## Conclusion

We believe that the CATT_5_/G have a protective effect on endometriosis. As well, increased repetitions of CATT and C allele in *MIF* promoter were associated with increased susceptibility to endometriosis in our population and this was related to transcriptional activity of *MIF*. These findings provide the first insight that *MIF* promoter polymorphisms may have a significant effect on susceptibility towards endometriosis; however, further studies are required to determine contribution of MIF to development of endometriosis.

## References

[1] Bulun SE, Zeitoun K, Takayama K, Noble L, Michael D, Simpson E (1999). Estrogen production in endometriosis and use of aromatase inhibitors to treat endometriosis. Endocr Relat Cancer.

[2] Burney RO, Giudice LC (2012). Pathogenesis and pathophysiology of endometriosis. Fertil Steril.

[3] Giudice LC (2010). Endometriosis. N Engl J Med.

[4] Sampson JA (1927). Peritoneal endometriosis due to the menstrual dissemination of endometrial tissue into the peritoneal cavity. Am J Obstet Gynecol.

[5] Kyama CM, Debrock S, Mwenda JM, D’Hooghe TM (2003). Potential involvement of the immune system in the development of endometriosis. Reprod Biol Endocrinol.

[6] Javeed A, Zhao Y, Zhao Y (2008). Macrophage-migration inhibitory factor: role in inflammatory diseases and graft rejection. Inflamm Res.

[7] Larson DF, Horak K (2006). Macrophage migration inhibitory factor: controller of systemic inflammation. Crit Care.

[8] Calandra T, Roger T (2003). Macrophage migration inhibitory factor: a regulator of innate immunity. Nat Rev Immunol.

[9] Renner P, Roger T, Calandra T (2005). Macrophage migration inhibitory factor: gene polymorphisms and susceptibility to inflammatory diseases. Clin Infect Dis.

[10] Kats R, Metz CN, Akoum A (2002). Macrophage migration inhibitory factor is markedly expressed in active and early-stage endometriotic lesions. J Clin Endocrinol Metab.

[11] Carli C, Metz CN, Al-Abed Y, Naccache PH, Akoum A (2009). Up-regulation of cyclooxygenase-2 expression and prostaglandin E2 production in human endometriotic cells by macrophage migration inhibitory factor: involvement of novel kinase signaling pathways. Endocrinology.

[12] Lin W, Chen S, Li M, Wang B, Qu X, Zhang Y (2010). Expression of macrophage migration inhibitory factor in human endometriosis: relation to disease stage, menstrual cycle and infertility. J Obstet Gynaecol Res.

[13] Kats R, Al-Akoum M, Guay S, Metz C, Akoum A (2005). Cycle-dependent expression of macrophage migration inhibitory factor in the human endometrium. Hum Reprod.

[14] Morin M, Bellehumeur C, Therriault MJ, Metz C, Maheux R, Akoum A (2005). Elevated levels of macrophage migration inhibitory factor in the peripheral blood of women with endometriosis. Fertil Steril.

[15] Mahdian S, Aflatoonian R, Yazdi RS, Yaghmaei P, Ramazanali F, Afsharian P (2015). Macrophage migration inhibitory factor as a potential biomarker of endometriosis. Fertil Steril.

[16] Donn RP, Ray DW (2004). Macrophage migration inhibitory factor: molecular, cellular and genetic aspects of a key neuroendocrine molecule. J Endocrinol.

[17] Donn R, Alourfi Z, Zeggini E, Lamb R, Jury F, Lunt M (2004). A functional promoter haplotype of macrophage migration inhibitory factor is linked and associated with juvenile idiopathic arthritis. Arthritis Rheum.

[18] Areeshi MY, Mandal RK, Dar SA, Jawed A, Wahid M, Lohani M (2017). MIF-173 G> C (rs755622) gene polymorphism modulates tuberculosis risk: evidence from a meta-analysis and trial sequential analysis. Sci Rep.

[19] Bae SC, Lee YH (2017). Circulating macrophage migration inhibitory factor levels and its polymorphisms in systemic lupus erythematosus: a meta-analysis. Cell Mol Biol (Noisy-le-grand).

[20] Illescas O, Gomez-Verjan JC, García-Velázquez L, Govezensky T, Rodriguez-Sosa M (2018). Macrophage migration inhibitory factor-173 G/C polymorphism: a global meta-analysis across the disease spectrum. Front Genet.

[21] Ma M, Tao L, Liu A, Liang Z, Yang J, Peng Y (2018). Macrophage migration inhibitory factor-794 CATT microsatellite polymorphism and risk of tuberculosis, a meta-analysis. Biosci Rep.

[22] Shi YY, He L (2005). SHEsis, a powerful software platform for analyses of linkage disequilibrium, haplotype construction, and genetic association at polymorphism loci. Cell Res.

[23] Li Z, Zhang Z, He Z, Tang W, Li T, Zeng Z (2009). A partition-ligationcombination-subdivision EM algorithm for haplotype inference with multiallelic markers: update of the SHEsis (http://analysis.bio-x.cn). Cell Res.

[24] Chekini Z, Yaran AP, Ansari-Pour N, Shahhoseini M, Ramazanali F, Aflatoonian R (2019). The novel gene-wide haplotype at the macrophage migration inhibitory factor (MIF) locus is associated with endometrioma. Eur J Obstet Gynecol Reprod Biol.

[25] Zhong XB, Leng L, Beitin A, Chen R, McDonald C, Hsiao B (2005). Simultaneous detection of microsatellite repeats and SNPs in the macrophage migration inhibitory factor (MIF) gene by thin-film biosensor chips and application to rural field studies. Nucleic Acids Res.

[26] Awandare GA, Martinson JJ, Were T, Ouma C, Davenport GC, Ong’echa JM (2009). MIF (macrophage migration inhibitory factor) promoter polymorphisms and susceptibility to severe malarial anemia. J Infect Dis.

[27] Grieb G, Merk M, Bernhagen J, Bucala R (2010). Macrophage migration inhibitory factor (MIF): a promising biomarker. Drug News Perspect.

[28] Donn R, Alourfi Z, De Benedetti F, Meazza C, Zeggini E, Lunt M (2002). Mutation screening of the macrophage migration inhibitory factor gene: positive association of a functional polymorphism of macrophage migration inhibitory factor with juvenile idiopathic arthritis. Arthritis Rheum.

[29] Baugh JA, Chitnis S, Donnelly SC, Monteiro J, Lin X, Plant BJ (2002). A functional promoter polymorphism in the macrophage migration inhibitory factor (MIF) gene associated with disease severity in rheumatoid arthritis. Genes Immun.

[30] Yang J, Li Y, Zhang X (2015). Meta-analysis of macrophage migration inhibitory factor (MIF) gene-173G/C polymorphism and inflammatory bowel disease (IBD) risk. Int J Clin Exp Med.

[31] Zhang X, Weng W, Xu W, Wang Y, Yu W, Tang X (2015). The association between the migration inhibitory factor− 173g/c polymorphism and cancer risk: a meta-analysis. Onco Targets Ther.

[32] Ku WC, Chiu SK, Chen YJ, Huang HH, Wu WG, Chen YJ (2009). Complementary quantitative proteomics reveals that transcription factor AP-4 mediates E-box-dependent complex formation for transcriptional repression of HDM2. Mol Cell Proteomics.

[33] Vallette-Kasic S, Pellegrini-Bouiller I, Sampieri F, Gunz G, Diaz A, Radovick S (2001). Combined pituitary hormone deficiency due to the F135C human Pit-1 (pituitary-specific factor 1) gene mutation: functional and structural correlates. Mol Endocrinol.

[34] Agarwal S, Cho TY (2017). Biochemical and structural characterization of a novel cooperative binding mode by Pit-1 with CATT repeats in the macrophage migration inhibitory factor promoter. Nucleic Acids Res.

[35] Yao J, Leng L, Sauler M, Fu W, Zheng J, Zhang Y (2016). Transcription factor ICBP90 regulates the MIF promoter and immune susceptibility locus. J Clin Invest.

[36] Veillat V, Sengers V, Metz CN, Roger T, Leboeuf M, Mailloux J (2012). Macrophage migration inhibitory factor is involved in a positive feedback loop increasing aromatase expression in endometriosis. Am J Pathol.

[37] Cao WG, Morin M, Metz C, Maheux R, Akoum A (2005). Stimulation of macrophage migration inhibitory factor expression in endometrial stromal cells by interleukin 1, beta involving the nuclear transcription factor NFκB. Biol Reprod.

